# Rheological Characterization and Printability of Polylactide (PLA)-Alumina (Al_2_O_3_) Filaments for Fused Deposition Modeling (FDM)

**DOI:** 10.3390/ma15238399

**Published:** 2022-11-25

**Authors:** Anton Smirnov, Anton Seleznev, Pavel Peretyagin, Ekaterina Bentseva, Yuri Pristinskiy, Ekaterina Kuznetsova, Sergey Grigoriev

**Affiliations:** 1 Laboratory of 3D Structural and Functional Engineering, Moscow State University of Technology “STANKIN”, Vadkovsky per. 1, Moscow 127055, Russia; 2 Spark Plasma Sintering Research Laboratory, Moscow State University of Technology “STANKIN”, Vadkovsky per. 1, Moscow 127055, Russia; 3 Scientific Department, A.I. Evdokimov Moscow State University of Medicine and Dentistry, Delegatskaya St., 20, p.1, Moscow 127473, Russia

**Keywords:** polymer-ceramic mixtures, rheological properties, extrusion, 3D printing, FDM/FFF

## Abstract

This article presents the study of the rheological properties and the printability of produced ceramic-polymer filaments using fused deposition method (FDM) 3D printing technology. Powder mixtures with an alumina content of 50 to 70 vol.% were fabricated by a wet processing route. A series of rheological experiments of the obtained mixtures were conducted in the temperature range from 200 to 220 °C for the commercial polylactide (PLA) powder and from 200 to 240 °C for ceramic-polymer, which corresponds to the recommended temperatures for 3D printing of commercial PLA filaments. The composition with the maximum content of alumina leads to a powdery material in which the molten polymer is insufficient to measure the rheological properties. In spite of this, the filaments were prepared from all the obtained mixtures with a tabletop single-screw extruder, the diameter and surface profile of which were analyzed. As the ceramic content increased, the diameter and surface roughness of the filaments increased. Therefore, it was only possible to print an object from a filament with the lowest ceramic content. However, the print quality of the 3D printed objects from the fabricated ceramic-polymer filament is worse (imperfect form, defects between layers) compared to the commercial PLA filament. To eliminate such defects in the future, it is necessary to conduct additional research on the development of printing modes and possibly modify the software and components of the 3D printer.

## 1. Introduction

Because of their excellent properties, ceramics are used in a wide variety of applications, including chemical, mechanical, electronic, aerospace, and biomedical engineering [1,2,3,4,5,6,7,8]. The properties that make them such versatile materials include high mechanical strength and hardness, good thermal and chemical stability, and viable thermal, optical, electrical and magnetic characteristics. The desired configuration of ceramic products is usually made from a mixture of powders with (or without) binder additives using conventional techniques, including injection molding, centrifugal molding, belt molding, uniaxial and isostatic pressing, etc. Moreover, to give products their final strength, compactness and density of their material, a complete synthesis of the required phases, including the formation of the size of crystalline grains, the state of their boundaries, and the sintering operation, is carried out. However, these technologies of product formation are characterized by a long duration and, accordingly, a high cost. In addition, although these methods are able to obtain products with improved properties, the possibility of obtaining products of complex configuration is limited by the technological features of the sintering processes, although intensive research has been conducted in this direction. In addition, the processing of blanks is extremely difficult because of the extreme hardness and natural brittleness of ceramics. Making each piece requires high costs and labor invested in the purchase of precision machining tools based on artificial diamond and achieving high surface quality and dimensional accuracy of the product is limited by the geometry of the tool used. Ceramics cannot be machined on bladed equipment, so expensive diamond turning and grinding operations are required to produce the final product. For this reason, the need to manufacture ceramic products of complex shape that require minimal additional machining is extremely urgent. Therefore, the introduction of 3D printing or additive technologies, involving the manufacture of a product according to a computer aided design (CAD) model, in the production of arbitrarily complex ceramic components, opens up completely new opportunities to solve the above-mentioned problems and challenges. According to ISO/ASTM 52900:2015, these technologies are divided into the following categories: vat photopolymerization, powder-bed fusion, material jetting, material extrusion, direct energy deposition, binder jetting, and sheet lamination. However, despite the existence of many methods of 3D printing, most of them do not allow rapid prototyping of ceramic components of arbitrary shapes. In addition, existing methods of 3D printing ceramics have many drawbacks, which primarily include expensive laser or electron-beam equipment and consumables for sintering or melting processes.

**Figure 1:**
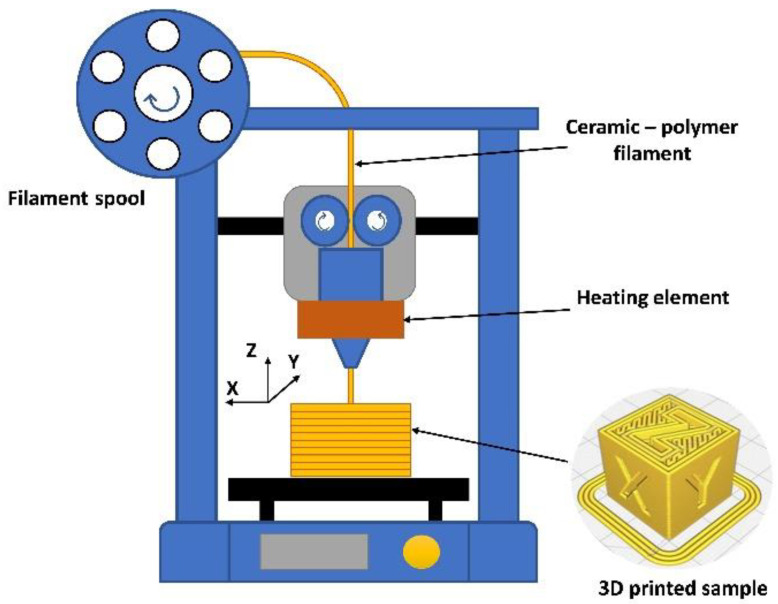
Schematic diagram of FDM/FFF-3D printing.

Compared to other additive manufacturing (AM) processes, the material extrusion method is the most widely used, especially when working with polymers and thermoplastic composites [9]. In addition, the equipment used for this method is inexpensive and very easy to operate [10,11,12]. Therefore, the main advantage of the material extrusion method, which involves fused deposition modeling (FDM) or fused filament fabrication (FFF), is the rapid and cheap reproduction of standard components or prototypes with a variety of polymeric materials. Both techniques involve creating three-dimensional objects by applying successive layers of molten material, repeating the contours of the digital model (Figure 1).

The main difference is the historical background of these two terms. The FDM is a proprietary, 3D printing technology developed and coined by Stratasys in 1989 [13]. The Stratasys’ patents for FDM technology were about to expire only in 2009, but in 2005 a new project was started with the name RepRap (replicating rapid prototyper) whose members came up with their own synonymous term (FFF) that can be used by anyone without restriction [14]. However, in spite of the use of the material extrusion method for fabrication of ceramic parts that are highly attractive, research data in this field are still scarce, although recently it has experienced considerable interest. As an example, Weng et al. produced acrylonitrile butadiene styrene (ABS)/montmorillonite (OMMT) filaments for 3D printing by melt extrusion. Printed samples with OMMT increased the mechanical properties compared with polymer specimens [15]. Singh et al. fabricated a Nylon-6 filament reinforced by alumina and studied the mechanical properties of the obtained wire [16]. Kalita et al. developed particulate-reinforced polymer-ceramic composites by high shear mixing of polypropylene (PP) polymer and tricalcium phosphate (TCP) ceramic and controlled porosity scaffolds were fabricated via the fused deposition process [17]. Iyer et al. reported that proper FDM parameters resulted in dense, homogeneous, near-net-shape silicon nitride (Si_3_N_4_), with microstructures and mechanical properties similar to conventionally processed material [18]. Khatri et al. presented a process for the development, characterization, and correlation of ABS/up to 35 vol.% barium titanate polymer-ceramic composites produced by fused deposition modeling [19]. Allahverdi et al. fabricated a number of filaments such as lead–zirconate–titanate (PZT), lead–magnesium–niobate (PMN), alumina (Al_2_O_3_), and bismuth titanate (BiT), which were used in the FDM process [20]. Nötzel et al. developed a ceramic-polymer composite as filament material that can be printed on a low-cost, fused-filament-fabrication desktop printer, even with very small nozzle sizes enabling very small geometric feature sizes [21]. Thin ceramic (calcium phosphate) periodic structures with a spatial resolution of <100 µm and precise dimensions and intricate hierarchical structure have been successfully fabricated using the FDM by Yang et al. [22]. In [23], the use of a unit with four extruders to produce multilayer sensors with different types of soft and hard ceramics in the same layer was shown.

It is well known that various characteristics of the ceramic feedstock affect printing, such as the size of ceramic particles, their distribution in the filament, suitable rheology for the applied process to ensure shape retention and good welding of the filaments, the ceramic/binder/additive ratio, viscosity, and the flexibility of the filament [24,25].

Based on the above information, the purpose of this work is to produce highly filled ceramic-polymer mixtures, to study the rheological behavior of the prepared mixtures, to extrude filaments from them and to print the objects from the fabricated filaments.

## 2. Materials and Methods

### 2.1. Preparation of Ceramic-Polymer Mixtures

Commercial polylactide (PLA) powder (eSun Ltd., Shenzhen, China) and alumina (Al_2_O_3_) powder (Plasmotherm Ltd., Moscow, Russia) with median particle size around 35 and 30 µm, respectively, were used as feedstock in this work. Alumina was chosen due to its superior hardness, high temperature resistance, and relative low cost. At the same time, PLA has such advantages as biodegradability and biocompatibility, cheapness, and dimensional stability (low shrinkage). In addition, PLA allows printing complex shapes and is easy to print on a standard 3D printer. To obtain filaments in this study, compositions with 50, 60, and 70 vol.% content of the ceramic component were chosen. Mixing of ceramics and polymers was carried out in a PM 100 planetary mill (Retsch, Haan, Germany). The initial raw materials were stirred in a grinding bowl with alumina balls (d = 3 mm) in distilled water for 2 h at a speed of 200 rpm. After homogenization, the mixture was dried for 24 h in a vacuum oven VO 400 (Memmert GmbH, Büchenbach, Germany) at 90 °C, and then sieved on a vibratory sieve shaker AS 200 (Retsch, Haan, Germany).

### 2.2. Microstructural Characterization and X-ray Diffraction (XRD)

Phase identification of ceramic-polymer compositions was carried out by X-ray Dif-fraction in an Empyrean diffractometer (PANalytical, Almelo, The Netherlands) with radiation source Cu–Kα (λ = 1.5405981 Å) operated with an intensity of 30 mA at 40 kV in the 2θ angle range from 10 to 70°. The analysis was carried out with a scanning speed of 0.06/min and a step size of 0.05. The microstructure and powder morphology of the initial powders as well as elemental composition of obtained filaments were studied using a scanning electron microscope (SEM) VEGA 3 LMH (Tescan, Brno, Czech Republic) equipped with a module for energy dispersive X-ray spectroscopy (EDS) analysis. The diameter of the obtained filaments and the spatial dimensions of the printed samples were measured with an absolute digimatic caliper 100 (Mitutoyo, Kawasaki Japan). The study of the relief of the filament surface was carried out on a stereomicroscope SZ61 (Olympus, Tokyo, Japan), and the roughness values were obtained on a desktop SEM G2 Pro X (Phenom, Eindhoven, The Netherlands).

### 2.3. Fourier Transform Infrared Characterization

Fourier transform infrared (FTIR) spectra of the prepared mixtures were measured (Vertex 70 spectrometer, Bruker AXS Inc., Madison, WI, USA) in the wavenumber range from 500 cm^−1^ to 4000 cm^−1^ (transmission mode, resolution 2 cm^−1^, 120 scans per sample). For this purpose, potassium bromide (KBr) pellets (diameter 1.0–1.3 cm) of each powder sample were prepared using a uniaxial press [26].

### 2.4. Rheological Characterization

Rheological studies of the prepared mixtures were carried out on a Haake MARS rotational rheometer (Thermo Fisher Scientific Inc., Waltham, MA, USA) using a plane-plane configuration, a plate diameter of 20 mm, and a gap of 1 mm. Rheological behavior was investigated according by the following modes: (1) frequency dependence of the dynamic modulus components at a strain amplitude equal to 1%, strain frequency varied from 0.1 to 100 Hz; (2) creep experiments at constant stress of 10, 100, and 500 Pa for unfilled (without ceramic component) PLA followed by load removal (“recovery” stage); (3) viscosity dependence on shear rate (shear rate range was stepwise increased from 0.001 to 10 s^−1^ with holding at each step for 30 s). Prior to testing, the specimens were dried at 80 °C for 6 h in a vacuum oven VO 400 (Memmert GmbH, Büchenbach, Germany).

### 2.5. Differential Scanning Calorimetry and Thermogravimetric Analysis

To study the thermal transitions and determine the decomposition temperature of the studied specimens, differential scanning calorimetry (DSC) and thermogravimetric analysis (TGA) were carried out. The first study was performed on a DSC3+ analyzer (Mettler Toledo, Nänikon, Switzerland), with a liquid nitrogen cooling system, equipped with an HSS9+ sensor. Method program: heating–cooling–reheating at a constant rate of 10 K/min, in the range from 0 to 250 °C, in an N_2_ atmosphere, with a flow rate of 50 mL/min. The sample was placed in a 40 µL alumina crucible. TGA analyses were performed on TGA/DSC3+ synchronous thermal analysis equipment (Mettler Toledo, Nänikon, Switzerland) equipped with an HSS2 sensor. Tests were performed in a heating mode at a constant rate of 10 K/min, ranging from 25 to 1000 °C, in an air atmosphere, at a flow rate of 50 mL/min. A 70 µL Al_2_O_3_ crucible was used.

### 2.6. Extrusion of Filaments and 3D Printing

Prior to extrusion, the obtained ceramic-polymer mixtures were pre-pressed on an OPAL 480 hot mounting press (QATM, Mammelzen, Germany) for 45 min at a pressure of 175 bar and a temperature of 185 °C. The obtained pellets were then loaded into the Wellzoom (Shenzhen Mistar Technology Co., Shenzhen, China) tabletop single-screw extruder hopper. The ceramic-polymer filaments were produced at temperatures between 200 °C and 240 °C and with a nozzle diameter of 1.75 mm. Specimens in the shape of cubes were printed from extruded experimental ceramic-polymer filaments using a Black Widow 3D printer (Tevo 3D, Zhanjiang, China) under the following conditions: nozzle temperature 220 °C, table heating temperature 100 °C, nozzle diameter 0.8 mm, layer height 0.4 mm, filling 100%.

## 3. Results and Discussion

**Figure 2:**
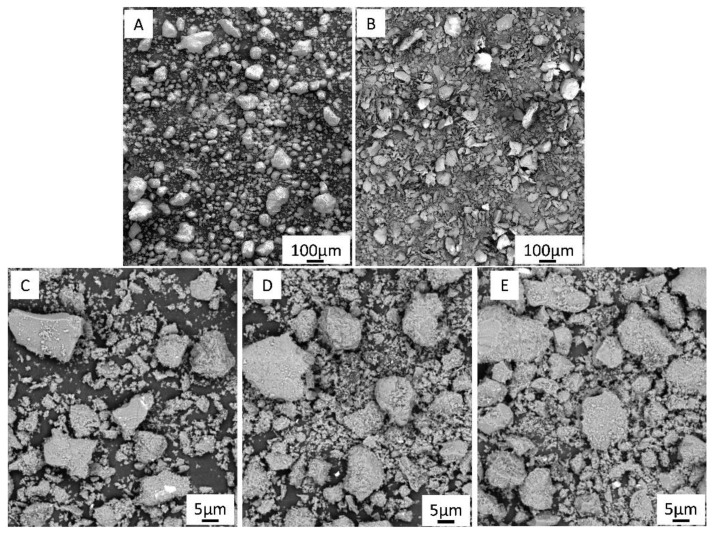
SEM micrographs of raw alumina (**A**), PLA (**B**) and ceramic-polymer mixtures with 50 (**C**), 60 (**D**) and 70 (**E**) vol.% of Al_2_O_3_.

Figure 2 shows the microstructure of the initial raw powders and ceramic-polymer mixtures with different content of the ceramic component.

**Figure 3:**
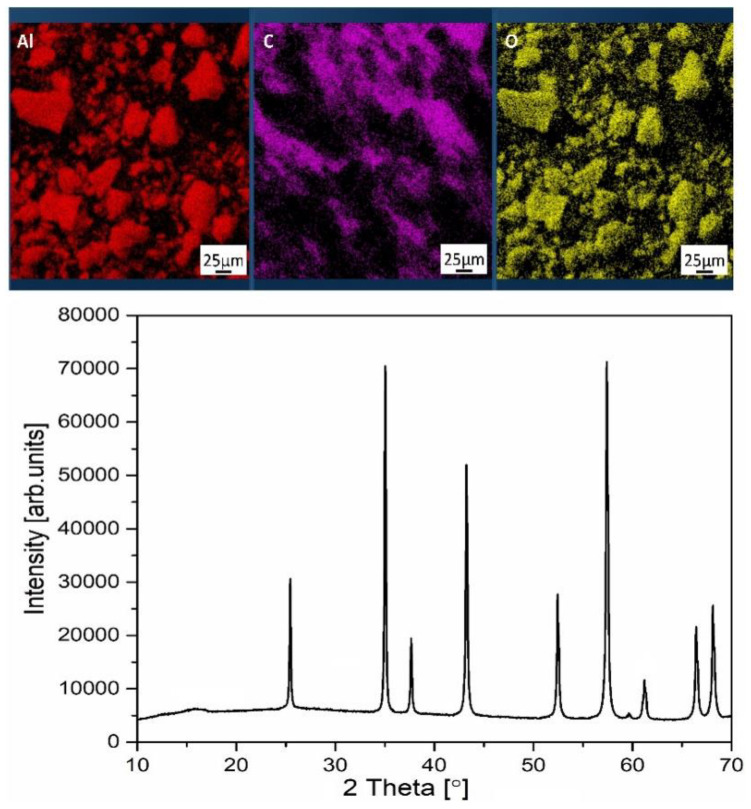
Representative SEM–EDS elemental distribution maps for aluminium, carbon and oxygen (**top**) and XRD pattern (**bottom**) for ceramic-polymer mixture with 50 vol.% of Al_2_O_3_.

The study of the microstructure of the alumina raw powder showed the presence of large agglomerates of ceramic particles, which have a clear non-spherical shape. The initial powder of PLA, in turn, is represented mainly by flake-shaped particles but there are also large inclusions of irregular shape. As a result of mixing in a planetary mill, there was a partial destruction of aluminum oxide agglomerates and grinding of polylactide powder (Figure 2C–E). Both raw materials lost their original form. The figure shows that the mixture is dominated by coarse aluminum oxide particles of irregular shape, 5–26 microns in size, with small particles of 1 to 5 microns of plastic adhered to them. The non-spherical shape of the raw material particles may cause difficulties with further extrusion of the ceramic-polymer filaments. Representative SEM–EDS elemental distribution maps and X-ray diffraction pattern corresponding to the ceramic-polymer mixture with 50 vol.% of Al_2_O_3_ are shown in Figure 3. The EDS and XRD studies show that no contaminants or new phases were detected during preparation of the mixture. A broad amorphous peak from PLA was detected around 16.8° that confirms amorphous microstructure of the PLA.

**Figure 4:**
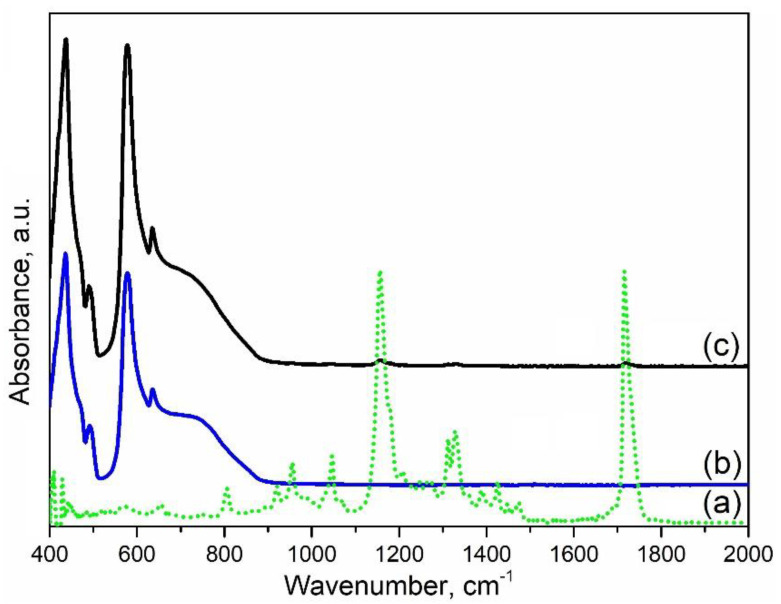
FTIR spectra of raw powders of PLA (**a**), alumina (**b**) and 50Al_2_O_3_/50PLA mixture (**c**).

The FTIR spectra of the raw powders and the 50Al_2_O_3_/50PLA mixture are shown in Figure 4. The characteristic peaks of aluminum oxide and polylactide are clearly visible on the graph, even in the sample with minimal ceramic content. No additional elements and compounds were detected. Thus, there was no contaminations or appearance of the new phases during the mixing process.

**Figure 5:**
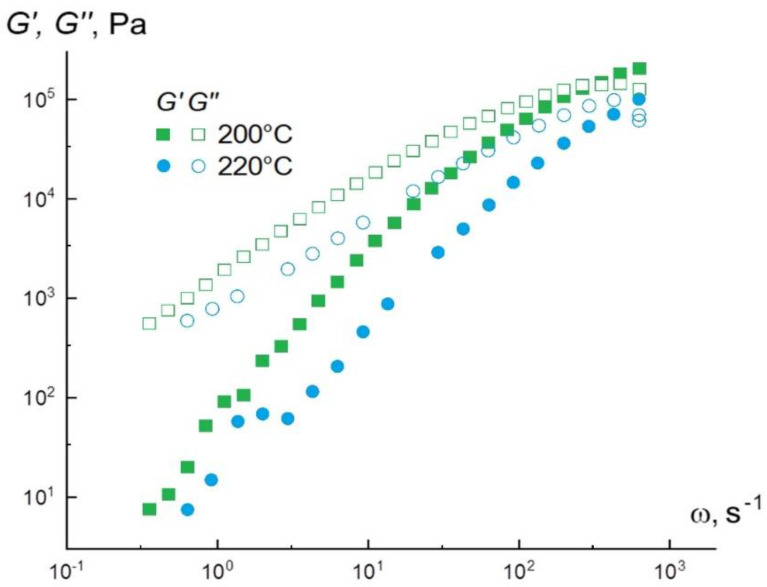
Frequency dependences of elastic and loss moduli for polylactide at different temperatures.

To obtain interpretable data on the viscoelasticity of materials, measurements must be made in the linear viscoelastic range. Figure 5 shows the frequency dependence plots of elastic and loss moduli for the PLA powder at temperatures of 200 °C and 220 °C.

**Figure 6:**
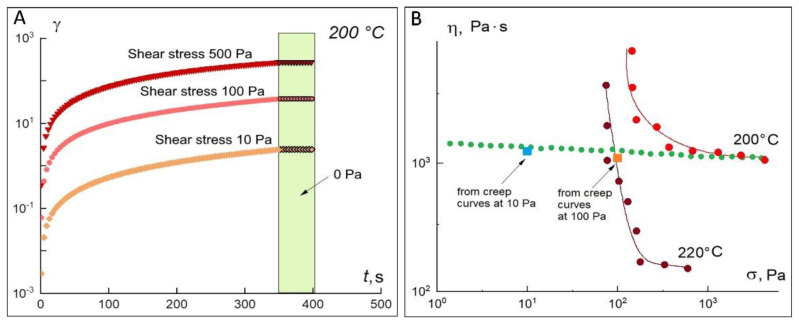
Shear stress-time dependence of polylactide at constant stress in “loading-unloading” mode at 200 °C (**A**). Viscosity versus shear stress for PLA at different temperatures, as well as viscosity values calculated from creep curves at different loads (**B**).

The values obtained show that, practically over the entire range of angular frequencies, the loss modulus G″ exceeds the storage modulus G′. This testifies to the viscous-flow state of the material under the given deformation conditions. The crossover point is in the upper frequency range and is 145 × 10^3^ Pa at 200 °C. In addition, they were the calculated tangents of the slope angle of the frequency dependences of the modules at 200 °C, which are: for G′ = 1.8, for G″ = 1, and at 220 °C for G′ = 1.7, and for G″ = 0.9. The found values practically correspond to the theory for the linear region of viscoelasticity (G′~ ω^2^ and *G*″~ω). Molten polylactide is a “living” material that changes its properties over time, so the sample should be deformed as quickly as possible and not left heated for too long. When exposed to high temperatures, the melt begins to darken in places where it contacts the air. Figure 6A demonstrates the creep curves at 200 °C obtained in the loading mode (σ = const) with subsequent unloading (or restoration), i.e., at σ = 0. If the material is a solid, it develops only elastic deformations, which drop to zero when the load is removed. If the material is a liquid, the strain achieved as a result of loading is stored in the material during unloading. Thus, it follows from Figure 6A that PLA is a viscoelastic fluid.

**Figure 7:**
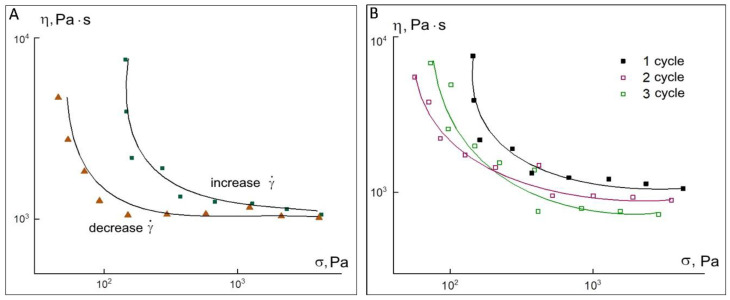
Dependences of polylactide viscosity on shear stress in shear rate increasing and decreasing mode (**A**). Results of repeated measurements of the flow curve of structured polylactide melt at cyclic increase and decrease in shear rate (for clarity, the curves are shown only in the shear rate increase mode) (**B**).

In constant-load mode, the viscosity values were calculated from the angle of the slope of the rectilinear sections of the observed dependences (in linear coordinates) in the time interval greater than 150 s, which are plotted on the flow curves obtained in the steady-state shear mode. When the load is removed (the so-called recovery region is marked in green in Figure 6A), the stress is practically not reduced (the maximum reduction is only 0.2%), indicating that at a given stress, all the energy supplied is spent on viscous flow. Figure 6B shows two flow curves corresponding to a temperature of 200 °C. In steady-state shear tests, in some experiments performed on a “fresh” specimen, the existence of a yield strength of the order of 100 Pa is observed. At the same time, the above frequency dependences of the elastic modulus and yield curves indicate that there is no stable structure that could exist up to a shear stress of the order of 100 Pa. This strange behavior can be explained by the fact that PLA is a crystallizing polymer, in which even at temperatures above the melting point a time-decaying structure can exist. The yield point curves (purple and red curves) appear in those experiments where the time between loading and the start of the experiment was minimal and the sample did not have time to fully melt before the test started. The green curve in Figure 6B, as well as the viscosity values calculated from the yield experiments, correspond to a fully melted polymer with a long dwell time before starting the test. As can be seen from the figure, these values are virtually independent of shear rate or stress, i.e., the melt behaves as a Newtonian fluid. In experiments carried out in the regime of increasing and then decreasing shear rate on an incompletely melted sample, the structure is restored (Figure 7A). This indicates the thixotropic behavior of the polylactide under the conditions of the preservation of the residual crystal structure of the insufficiently warmed sample, which should be kept in mind when using this polymer for 3D printing.

**Figure 8:**
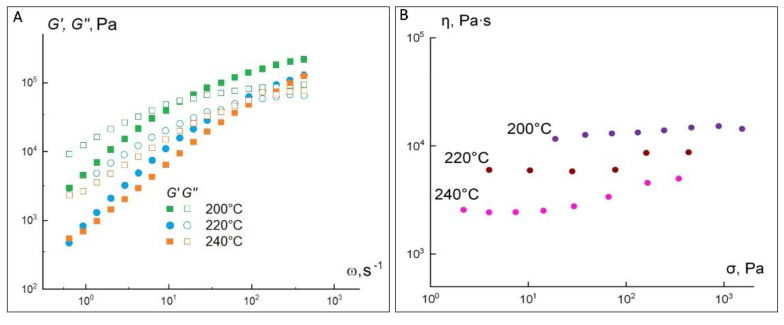
Frequency dependences of elastic and loss moduli (**A**) and viscosity versus shear stress for the 50Al_2_O_3_ composition at different temperatures (**B**).

As can be seen from these data, the curves smooth out with each new measurement cycle (Figure 7B) and should in the limit come to the Newtonian dependence shown in Figure 6B. Thus, the polylactide melt in the investigated temperature range is a viscoelastic liquid with a viscosity of about 10^3^ Pa·s at 200 °C. As the temperature increases to 220 °C, the melt viscosity decreases to 160 Pa·s. For the 50 Al_2_O_3_ ceramic-polymer mixture, the set of rheological properties is markedly different from the properties of the PLA powder. Figure 8 shows the frequency dependences of the elastic modulus G′ and loss modulus G″ at temperatures of 200, 220 and 240 °C for a composition containing 50 vol.% of alumina.

In contrast to PLA, the frequency curves of the 50Al_2_O_3_/50PLA composition already intersect at a frequency of about 60 s^−1^, above which elastic deformations predominate. The crossing point at 200 °C corresponds to an elastic modulus value of 8.75 × 10^4^ Pa, whereas for PLA it lies at 14.5 × 10^4^ Pa. Above this value, the elastic component of the dynamic modulus dominates the loss modulus, indicating that the elasticity of the material becomes dominant. It is expected that as the temperature increases, the moduli values will decrease and the crossover point will shift toward higher angular frequencies. Table 1 summarizes the viscoelastic properties of the investigated compound.

Table 1 demonstrates that the angular coefficients of the frequency dependences of the modulus of elasticity decrease in comparison with the polymer. Moreover, they become comparable in magnitude with the corresponding values for the loss modulus. Most likely, with 50 vol.% Al_2_O_3_, a framework can be formed, which causes additional structuring of the system. Under steady-state shear conditions, a composition with 50 vol.% Al_2_O_3_ behaves as a Newtonian fluid with a viscosity of 1.5 × 10^4^ Pa·s at 200 °C (Figure 8B), which is about 15 times the viscosity of PLA. This increase in viscosity is due to the presence of the ceramic particles and the adsorption of polymer macromolecules on it. It is noteworthy that the introduction of the ceramic phase does not lead to deviations from the Newtonian flow at 200 °C (Figure 8B), although this melt is more structured according to the viscoelasticity data. It can be assumed that the resulting structure is sufficiently strong and does not collapse in the stress range in question. This is also evidenced by the fact that both at 220 and 240 °C (Figure 8B) there is an increase in viscosity with increasing shear stress, which was reproduced in repeated experiments. Perhaps, the observed effect is related either to the improvement of the framework formed by the aluminum oxide particles or to a possible chemical interaction between the polymer and the ceramic component. It can thus be said that the ceramic-polymer mixture containing 50 vol.% of aluminum oxide is a viscoelastic Newtonian fluid with a viscosity significantly higher than the PLA powder. This result, as well as the decrease in the angular coefficients of the frequency dependences of the loss and elastic moduli, indicates the formation of a sufficiently strong structure in the composite. At the same time, the presence of 50 vol.% of ceramic almost completely suppresses crystallization, so that the flow curves (in contrast to PLA powder) lack the yield strength. At 220 and 240 °C, an increase in viscosity with increasing shear rate is observed, which is probably related to the appearance of strong contacts between the aluminum oxide particles or to a specific interaction between the polymer and the ceramic component.

**Figure 9:**
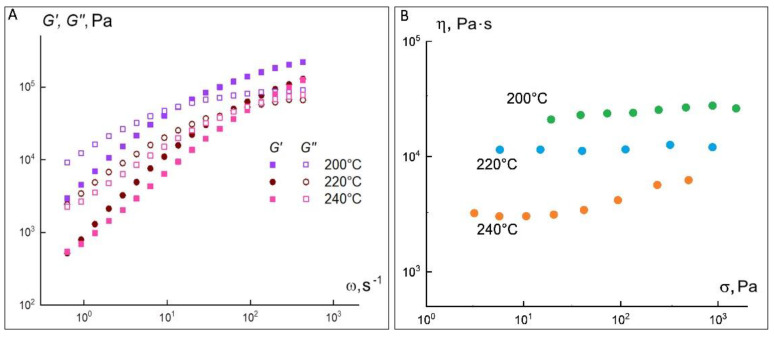
Frequency dependences of elastic and loss moduli (**A**) and viscosity versus shear stress for the 60Al_2_O_3_/40PLA composition at different temperatures (**B**).

The complex of the obtained rheological properties of the ceramic-polymer composition containing 60 vol.% of alumina differs from the properties of polylactide and the 50Al_2_O_3_/50PLA composition. Figure 9A shows the frequency dependences of the elastic and loss moduli G′ for this composition at different temperatures of 200, 220 and 240 °C.

In contrast to PLA and the 50Al_2_O_3_/50PLA composition, the frequency curves intersect already at a frequency of about 20 s^−1^, above which elastic deformations predominate. The crossing point at 200 °C corresponds to an elastic modulus value of 5.4 × 10^4^ Pa, whereas for PLA it lies at 14.5 × 10^4^ Pa. Above this value, the elastic component of the dynamic modulus dominates the loss modulus, indicating that the elasticity of the material prevails. As the temperature increases, the moduli values expectedly decrease, and the point of intersection shifts to the region of higher angular frequencies. The viscoelastic properties of the 60Al_2_O_3_/40PLA composition are presented in Table 2.

The angular coefficients of the frequency dependences of the elastic modulus significantly decrease in comparison with polylactide and the 50Al_2_O_3_/50PLA composition as indicated in Table 2. Moreover, they become comparable in value to the corresponding values for the loss modulus. Apparently, when 60 vol.% of alumina is added, a framework is formed over the aluminum oxide particles, which causes additional structuring of the system, as in the case of the 50Al_2_O_3_/50PLA composition.

Figure 9A shows the plots of viscosity versus the shear stress at various temperatures of 200, 220 and 240 °C for the 60Al_2_O_3_/40PLA composition. Under steady-state shear conditions, the above composition behaves as a Newtonian fluid with a viscosity of 2.4 × 10^4^ Pa·s at 200 °C, which is about 20 times the viscosity of polylactide. It should be noted that the introduction of 60 vol.% of Al_2_O_3_ does not lead to deviations from the Newtonian flow at 200 and 220 °C. It can be assumed that the structure is sufficiently strong and does not collapse in the stress range in question. This is also evidenced by the fact that, at 240 °C, there is an increase in viscosity with the increasing shear stress, which was reproduced in repeated experiments.

**Figure 10:**
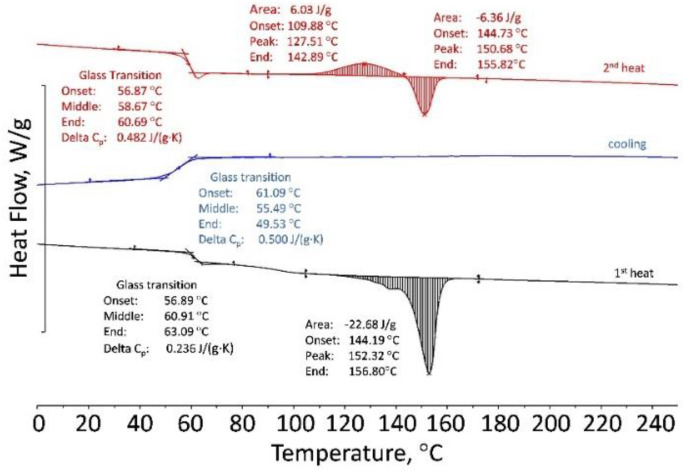
DSC curves of PLA.

**Figure 11:**
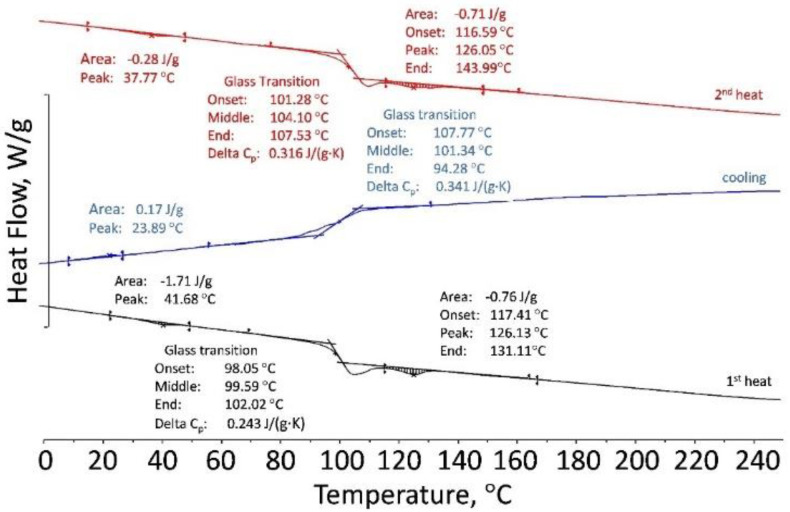
DSC curves of the 50Al_2_O_3_/50PLA composition.

**Figure 12:**
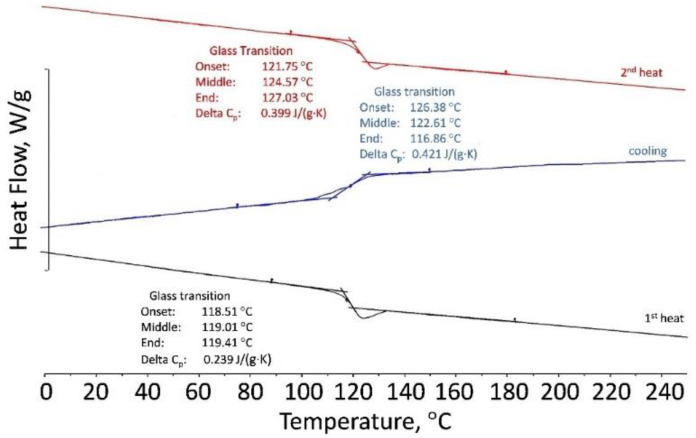
DSC curves of the 60Al_2_O_3_/40PLA composition.

In order to study and explain the viscosity-shear stress behavior for PLA, a DSC and TGA analysis was performed on the 50Al_2_O_3_/50PLAand 60Al_2_O_3_/40PLA compositions at different temperatures. Results of DSC showed that the polylactide sample is a “live” crystallizing polymer melting at 145–155 °C. Rapid cooling at a rate of 10 K/min revealed only the glass transition point at 50–61 °C. At the second heating in the temperature range from 110 to 140 °C, an exo-peak is observed, corresponding to the so-called “cold” crystallization followed by melting at 150 °C (Figure 10). The 50Al_2_O_3_/50PLA composition appreciably differs from polylactide in its properties: crystallinity is suppressed and glass transition temperature (T_g_) shifts to the area of higher temperatures T_g_ = 101–108 °C (Figure 11). Studies of the 60Al_2_O_3_/40PLA showed complete suppression of crystallinity and a shift of the glass transition temperature to higher temperatures T_g_ = 122–127 °C (Figure 12).

**Figure 13:**
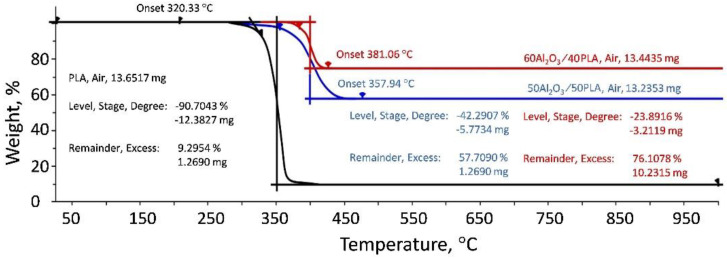
TGA curves of PLA, 50Al_2_O_3_/50PLA and 60Al_2_O_3_/40PLA compositions.

Figure 13 shows the results of the thermogravimetric analysis for polylactide and ceramic-polymer compositions with a ceramic content of 50 and 60 vol.%. The TGA results exhibited that the addition of alumina to polylactide shifted the degradation temperature to higher temperatures: 358 °C for the 50Al_2_O_3_/50PLA composition and 381 °C for the 60Al_2_O_3_ composition compared to 320 °C for PLA. Based on the results of the rheological properties study, it can be concluded that the ceramic-polymer compositions with 50 and 60 vol.% of alumina are viscoelastic Newtonian fluids with a viscosity significantly higher than that of polylactide. This fact, as well as the decrease in the angular coefficients of the frequency dependences of elastic and losses moduli, testifies to the formation of a sufficiently strong structure in the compositions. The introduction of aluminum oxide into polylactide at the level of 50–60 vol.% completely suppresses crystallization. At temperatures of 220 °C for the 50Al_2_O_3_ compositions and 240 °C for the 60Al_2_O_3_/40PLA compositions, an increase in viscosity with increasing shear rate is observed, which is probably related to the appearance of strong contacts between the aluminum oxide particles or to a specific interaction between the polymer and the ceramic component.

**Figure 14:**
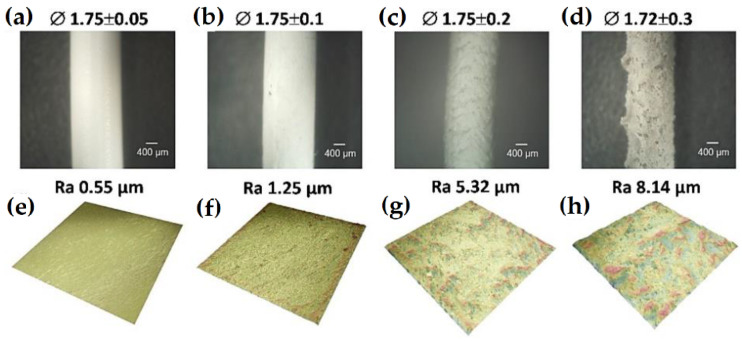
Optical photographs, diameter (**∅**), surface topography and measured roughness (***Ra***) of commercial polylactide (**a**,**e**), 50Al_2_O_3_/50PLA (**b**,**f**), 60Al_2_O_3_/40PLA (**c**,**g**) and 70Al_2_O_3_/30PLA (**d**,**h**) of the studied filaments.

It is worth mentioning that increasing the aluminum oxide content to 70 vol.% results in a powdery material in which the molten polymer is not sufficient to obtain a homogeneous sample to measure rheological properties. Despite the fact that it was not possible to study the rheological properties of the 70Al_2_O_3_/30PLA composition, it was decided to extrude all the fabricated ceramic-polymer mixtures. Filaments with less ceramic content were easier to extrude, had a smoother topography, and were lighter in color (Figure 14). A sample of commercial polylactide filament (Bestfilament, Moscow, Russia) was also examined for comparison with the produced filaments. The sample of the commercial PLA filament had the lowest roughness values of Ra = 0.55 µm and diameter value of 1.75 ± 0.05 mm. The 50Al_2_O_3_/50PLA filament was flexible, visually intact, and had a relatively smooth surface topography of Ra = 1.25 µm. This filament had insignificant deviations from the shape along its length, with a diameter value of 1.75 ± 0.1 mm. The 60Al_2_O_3_/40PLA sample had a grayer shade and a rougher (Ra = 5.32 µm) surface topography and slightly larger values of diameter than the 50Al_2_O_3_/50PLA filament. The 70Al_2_O_3_/30PLA filament was extruded poorly, broke and clogged the extruder nozzle during extrusion. The sample turned out uneven with flaky relief, brittle, and had a grayer color. This filament had the largest variations in diameter and roughness. The pronounced rough surface relief of the samples with more than 60 vol.% ceramic content is most likely due to the increased content of the ceramic component, its nonspherical morphology (Figure 14d,h), and the formation of agglomerates during the preparation of the ceramic-polymer compositions.

**Figure 15:**
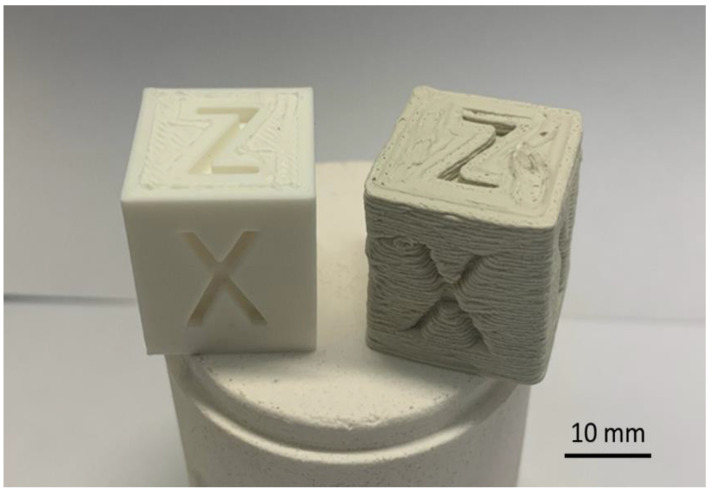
PLA (**left**) and 50Al_2_O_3_/50PLA (**right**) composition printed cubes.

In order to examine the printability of the obtained filaments, we tried to print a cube with a side equal to 20 mm. Unfortunately, it was not possible to perform 3D printing from filaments containing more than 50 vol.% of alumina, especially the 70Al_2_O_3_/30PLA composition, and even extrusion from the printer nozzle of these samples proved to be a very difficult process due to high viscosity. Figure 15 shows cubes printed by the FDM technique using commercial PLA and the fabricated 50Al_2_O_3_/50PLA filaments. After measuring the dimensions, it was found that the deviations of width (x), depth (y) and height (z) from the specified size (20 mm) were 0.1 and 0.4 mm, 0 and 0.3 mm and 0 and 0.1 mm for the PLA and 50Al_2_O_3_/50PLA printed cubes, respectively. The obtained values show that the quality of printing with the ceramic-polymer filament is still very much inferior to printing with the PLA plastic. The shape of the experimental sample turned out to be non-ideal, with obvious defects formed between the printed layers. Such results are due to the high viscosity of the composite material (15 times higher than the viscosity of PLA) and the use of non-optimized printing modes combined with the precision characteristics of the printer. To eliminate such defects in the future, it is necessary to conduct research on the development of printing modes and, possibly, to modify the software and components of the 3D printer.

## 4. Conclusions

In this work, a simple and solvent-free process of production of ceramic-polymer filaments with polylactide-based polymer matrix and high content of ceramic component from 50 to 70 vol.% is considered for printing by the FDM technique. The results of infrared spectroscopy of the ceramic-polymer mixtures confirmed that, with the selected method of filament production in their structure, there were no critical transformations and introduction of impurities into the composition. The results of the rheological studies of the obtained ceramic-polymer compositions showed that the content of the ceramic component up to 60 vol.% Al_2_O_3_ is satisfactory for printing by extrusion. However, it was only possible to print the object from the 50Al_2_O_3_/50PLA filament. Due to brittleness, irregular surface relief, and increased viscosity (15 times the viscosity of PLA), it was not possible to print samples of 60 and 70 vol.% filaments. Printing filaments with high ceramic content would require the use of an additive that reduces the viscosity, which would give the filaments flexibility during extrusion and allow for more accurate diameter values. At the moment, the print quality of the 3D printed objects from the 50Al_2_O_3_/50PLA filament are worse than that of the products made from the commercial PLA filament. Further research will focus on developing printing regimes, reducing defects in ceramic-polymer samples, finding parameters for removing the polymer binder and sintering the ceramic specimens with high density.

## Figures and Tables

**Table 1:** Viscoelastic behavior of the 50Al_2_O_3_/50PLA composition.

Temperature, °C	tg *G*′	tg *G*″	*ω,* s^−1^	*G,* Pa
200	1.25	0.75	55	114 × 10^3^
220	1.06	0.82	86	94 × 10^3^
240	1.18	0.68	150	108 × 10^3^

**Table 2:** Viscoelastic behavior of 60 Al_2_O_3_/40PLAcomposition.

Temperature, °C	tg *G′*	tg *G*″	*ω,* s^−1^	*G,* Pa
200	0.98	0.63	15	56 × 10^3^
220	1.17	0.81	45	43 × 10^3^
240	0.97	0.72	140	62 × 10^3^

## Data Availability

Not applicable.
